# P-41. Group A Streptococcus (GAS) Bloodstream Infection (BSI) in Patients Who Inject Drugs (PWID) : The Lesser of the Evils

**DOI:** 10.1093/ofid/ofaf695.270

**Published:** 2026-01-11

**Authors:** Lily Robinson, Andrea Molin, Stephanie Spivack

**Affiliations:** Temple University Hospital, Philadelphia, PA; Temple University Hospital, Philadelphia, PA; Temple University Health System, Philadelphia, PA

## Abstract

**Background:**

Cases of invasive GAS are rising nationally. The characteristics of GAS BSI are not well described in PWID and there is no standard treatment.

**Figure:**
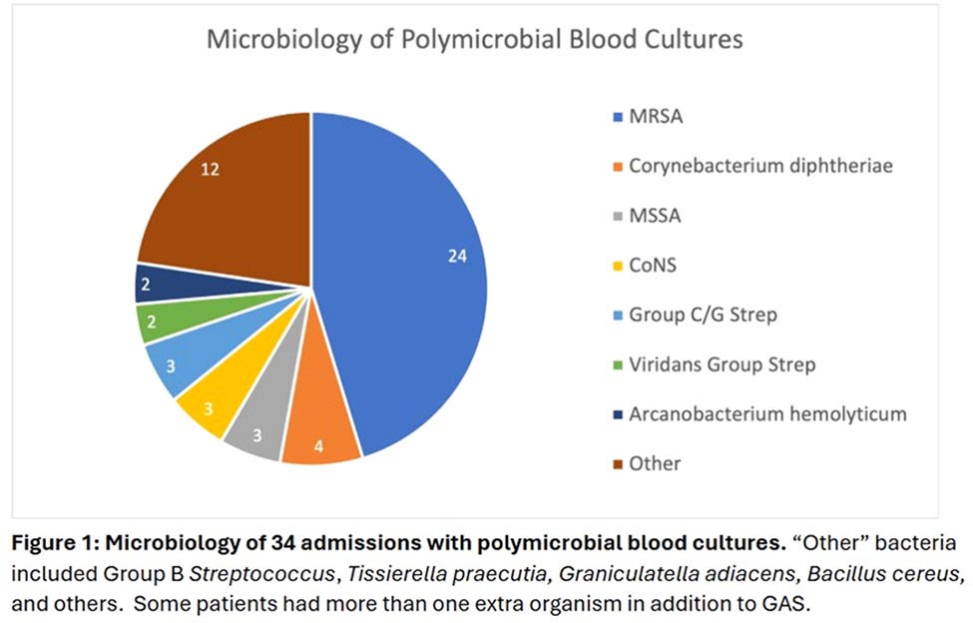

**Figure:**
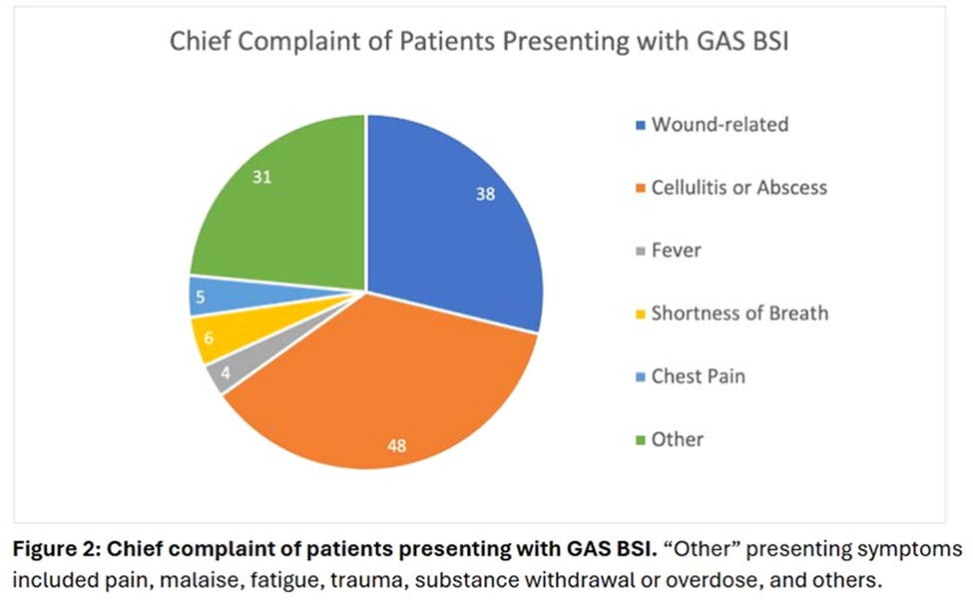

**Methods:**

This is an IRB-approved retrospective chart review of hospitalized PWID with positive blood cultures from March 2022 to March 2024.

**Figure:**
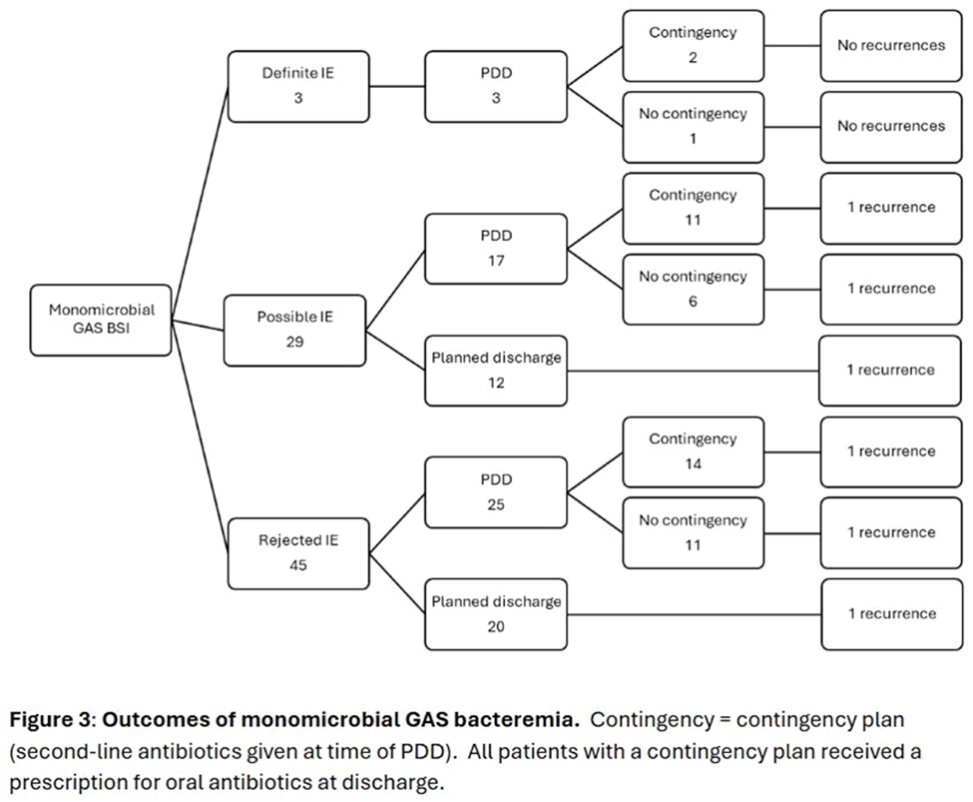

**Results:**

In total, 382 patients had 574 admissions with BSI. Of these, 102 patients (27%) had 111 (19%) admissions with GAS BSI. In this group, the median age was 38, 43% were female, 77% had unstable housing, and 57% had chronic wounds.

Of 111 GAS BSIs, 77 (69%) were monomicrobial and 34 (31%) were polymicrobial (Figure 1). In monomicrobial cases, the average time of blood culture positivity was 1.2 days; 70/77 had only 1 day of positive blood cultures, with a range of 2-4 days in the remaining 7 cases. Polymicrobial cases had an average of 2.6 days of blood culture positivity, ranging from 1 to 15 days. The average hospital length of stay was 7 vs. 15 days, respectively.

The most common presenting symptoms were cellulitis or abscess (43%) or wound-related (34%) (Figure 2). A transthoracic echocardiogram was completed in 65% of patients with monomicrobial and 80% of patients with polymicrobial BSI. By modified Duke criteria, 3% vs. 21% had definitive infective endocarditis (IE); 38% vs. 56% had possible IE; and 59% vs. 24% had rejected IE in the monomicrobial and polymicrobial groups, respectively.

For monomicrobial GAS BSI, 58% of discharges were patient-directed discharges (PDD). The recommended antibiotic plan included intravenous antibiotics in 30% of cases; oral antibiotics in 44%; dalbavancin in 1%; the remaining 28% had a PDD before a plan was implemented. For the 45 patients with monomicrobial GAS BSI who had a PDD, 27 were offered a contingency plan with oral antibiotics. We were able to confirm antibiotic completion in only 6 cases. 6/71 (8%) patients with monomicrobial GAS had a recurrent GAS BSI within 90 days (Figure 3), no mortalities were recorded, and no patients underwent surgery for endocarditis.

**Conclusion:**

PWID with monomicrobial GAS BSI had short durations of blood culture positivity, short hospital stays, and were rarely diagnosed with definite endocarditis or recurrent BSI. Outcomes were good despite high rates of PDD and low rates of antibiotic completion.

**Disclosures:**

All Authors: No reported disclosures

